# Flexural Strength of an Indirect Composite Modified with Single-Wall Carbon Nanotubes

**DOI:** 10.1055/s-0040-1721315

**Published:** 2021-05-31

**Authors:** Pedro Rogério Camargos Pennisi, Pedro Urquiza Jayme Silva, Fábio Scorsolini Valverde, Ticiane Campos Clemente, Vitória Cerri, Maria Eduarda Biaco, Rebeca Garcia Rosa Ferreira, Luiz Renato Paranhos, Eduardo Buozi Moffa

**Affiliations:** 1 School of Dentistry, Federal University of Uberlândia, Uberlândia/MG, Brazil; 2 School of Dentistry, University Center - UNIFAE, São João da Boa Vista/Sao Paolo, Brazil; 3 School of Physiotherapy, University Center - UNIFAE, São João da Boa Vista, Brazil; 4 College of Dentistry, University of Saskatchewan, Saskatoon, SK, Canada

**Keywords:** carbon nanotubes, composite resins, flexural strength

## Abstract

**Objectives** The low resistance to fracture has limited the use of indirect composite resins for dental restorations, particularly in regions that are exposed to strong occlusal forces. To overcome this issue, different types of reinforcement for composites have been proposed, one of which is carbon nanotubes (CNTs). The aim of this study was to evaluate the flexural resistance of one commercial indirect composite resin (Sinfony, 3M/ESPE) after incorporation of single-wall carbon nanotubes (SWCNTs; Sigma–Aldrich, Inc., St. Louis, Missouri, United States) with or without the silanization form.

**Materials and Methods** Specimens of composite resin were fabricated in a Teflon mold. The composite resin was prepared according to the manufacturer’s instructions ( *n* = 10 for each group), with SWCNTs in three concentrations.

**Statistical Analysis** The SWCNTs and SWCNT/SiO _2_ -ATES specimens were evaluated by transmission electron microscopy, and a flexural test was conducted according to the ISO 4049/2009. Flexural strength data in MPa were submitted to one-way ANOVA following Tukey ( *p* < 0.05).

**Results** The SWCNTs did not improve the flexural strength of indirect composite resin when compared with the control, independent of the concentration added ( *p* > 0.05). However, when SWCNTs and SWCNTs/SiO2-ATES were compared, the SWCNTs/SiO2-ATES showed higher values than the three concentrations of SWCNTs ( *p* < 0.05).

**Conclusion** The silanization process improves the SWCNTs strength proprieties, but the modification of chemical bonding between SWCNT and SWCNT/SiO _2_ -ATES modified resins, in different concentrations, did not improve the composite resin flexural strength.

## Introduction

Improvements in the properties of indirect composite resins (ICR) have been used in applications for inlays, onlays, and crowns. 1 Despite the significant development of these composites, ICR have shown unsatisfactory clinical performance, particularly in regions exposed to strong mastication forces, 4 and their relatively low resistance to fracture has limited the clinical usage. 4 5

A common technique to improve composite mechanical properties is the incorporation of fibers. 6 Different types of reinforcement for composites proposed have included carbon, graphite, glass, and Kevlar. 7 8 The effectiveness of fiber reinforcement is dependent on many variables: materials used, the quantity of fibers in the matrix, diameter, length, orientation of the fibers, and adhesion of fibers to the matrix polymer. 9 10 11 Some laboratory studies demonstrated that these reinforced resins had improved resistance to fracture, 12 13 while other studies have shown that there was no significant increased resistance to fracture. 14 15

The carbon nanotubes (CNTs) have excellent mechanical properties, electrical conductivity, rheological properties, thermal conductivity, stability, and flammability. 16 Therefore, the incorporation of CNTs can significantly enhance of such properties of polymer composite. 17

Various applications of CNTs fabrication of reinforced fibers and nanocomposites have been demonstrated in literature. 18 There are two types of CNT: single-wall carbon nanotubes (SWCNTs) and multi-wall carbon nanotubes (MWCNTs), 16 19 and various properties of these CNTs have been published. Single and multi-wall CNT vary significantly depending on the production, purification, suspension, filling, and functionalization. 19 In addition, good interfacial bonding between polymer and CNTs and the uniform dispersion of CNTs throughout the polymer matrix are critical in determining the mechanical properties of polymer CNTs composites. 20 According to the above considerations, the CNTs can be an excellent candidate to be incorporated in the laboratory-processed resin composite 16 to improve their flexural resistance, although there are no studies on this issue. Therefore, the purpose of this study was evaluated the flexural resistance of one commercial composite resin after incorporation of CNTs. Also, the effect of the silanization of CNTs on flexural resistance was studied. It is expected that a good dispersion and bonding of SWCNTs to dental resin can be achieved improving the mechanical properties.

## Materials and Methods

### Ethical Criteria

The present study followed the Krithikadatta et al 21 guidelines that suggests the elaboration of Checklist for Reporting In vitro Study Guidelines standardizing *in vitro* studies. This study did not require approval of ethical criteria, as it did not involve humans and animals directly and indirectly.

### Sample Qualification and Sample Calculation

In this study, the sample size was calculated based on previous studies 22 with a statistical power of 0.80, requiring 10 specimens per group.

**Fig. 1:**
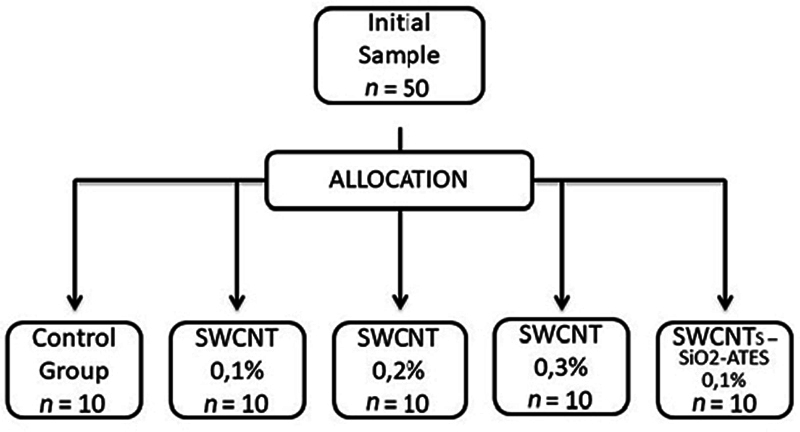
Allocation process flow chart.

The CNTs used were SWCNTs (Sigma–Aldrich, Inc.; St. Louis, Missouri, United States). The specimens were allocated in five groups to be analyzed ( Fig. 1 ): control group (without SWCNTs), SWCNTs at 0.1%, SWCNTs at 0.2%, SWCNTs at 0.3%, and SWCNTs-SiO _2_ -ATES at 0.1%.

### Silanization of Carbon Nanotubes

The silanization methodology followed Zhang et al. 22 First, the coating of Nano-SiO _2_ on CNT was prepared. This process involved sonication of SWNTs in an ethanol solution for 30 minutes, then incubation with 5 mmol/L APTES (3-aminopropyltriethoxysilane) for 30 minutes. The APTES-treated SWNTs were washed thoroughly with ethanol and were ultrasonically scattered in 20 mL ethanol for 30 minutes. Subsequently, 200 µL of concentrated NH _3_ .H _2_ O and 100 µL of TEOS (tetraethylorthosilicate) were added. After 30 minutes another 100μL of TEOS was added. For the desired SiO _2_ nanocoated SWNTs product _,_ the hydrolyzation process was conducted in ultrasonic conditions for 90 min. The samples were separated and washed with ethanol. The SWNTs-SiO _2_ were surface modificated with 5 mmol/L of organosilane ATES (allyltriethoxysilane) in ethanol, to produce the SWCNT-SiO _2_ -ATES. After washing with ethanol, the sample was dried at 120°C and cooled in a desiccator.

### Incorporation of Carbon Nanotubes in the Composite Resin

The composite resin was prepared according to fabricant instructions, without modifications for control group (without SWCNTs), and SWCNTs were added at 0.1, 0.2, and 0.3% (w/w) and SWCNTs-SiO _2_ -ATES at 0.1% (w/w). The resins were mechanically blended by spatulation to mimic clinical preparation.

After incorporations of SWCNTs and SWCNTs-SiO _2_ -ATES in different concentrations, specimens were fabricated in a Teflon mold (25 × 2 × 2 mm), prepolymerized using a visible-light-curing for 5 seconds with the Visio Alfa, and then polymerized in the Visio Beta device for 16 minutes under vacuum. All polymerization devices were used according to the manufacturer’s instructions (3M/ESPE). Wet silicon carbide papers (600-, 800-, 1,000-, and 1,500-grit) were used to polish and achieve a thickness of 2 mm; that was confirmed with a digital micrometer (Mitutoyo Corp.; Tokyo, Japan). The specimens were bathed in distilled water at 37°C for 24 hours.

### Data Collection and Analysis

The SWCNTs and SWCNT/SiO _2_ -ATES specimens were evaluated by transmission electron microscopy (TEM), and the flexural test was conducted according to the ISO 4049/2009 (ISO-Standards, 2009) performed in an EMIC DL 2000 assay machine connected to a 1,000 kgf loading cell with a speed of 0.5 mm/minute. The flexural strength was calculated using the formula:

where *F* is flexural strength, *Pf* is the maximum load (N), *L* is the distance between supports (mm), *W* is the sample width (mm), and *H* is the height of the sample (mm). Flexural strength data in MPa were submitted to analysis of variance (one-way ANOVA), following Tukey ( *p* < 0.05).

## Results

**Fig. 2:**
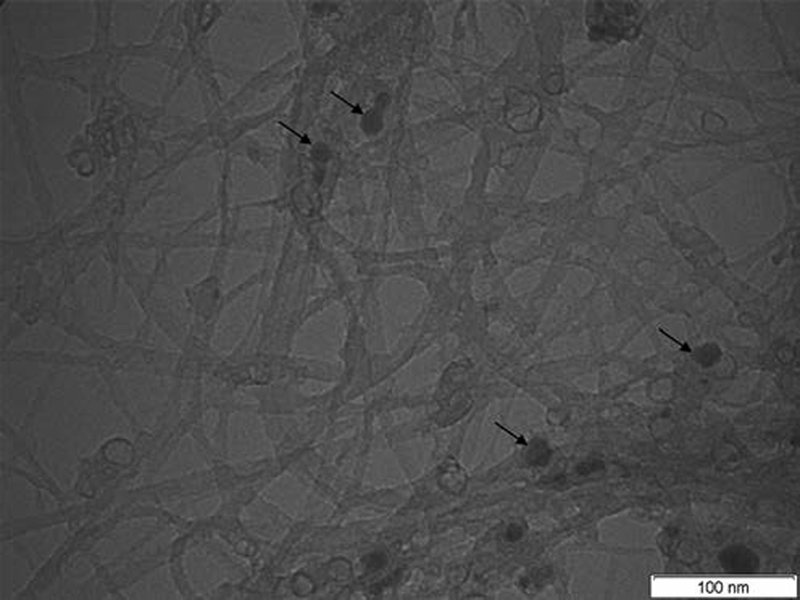
Transmission electron microscopyimage of original single-wall carbon nanotubes. Arrows show the presence of impurities, mainly amorphous carbon and metal nanoparticles.

**Fig. 3:**
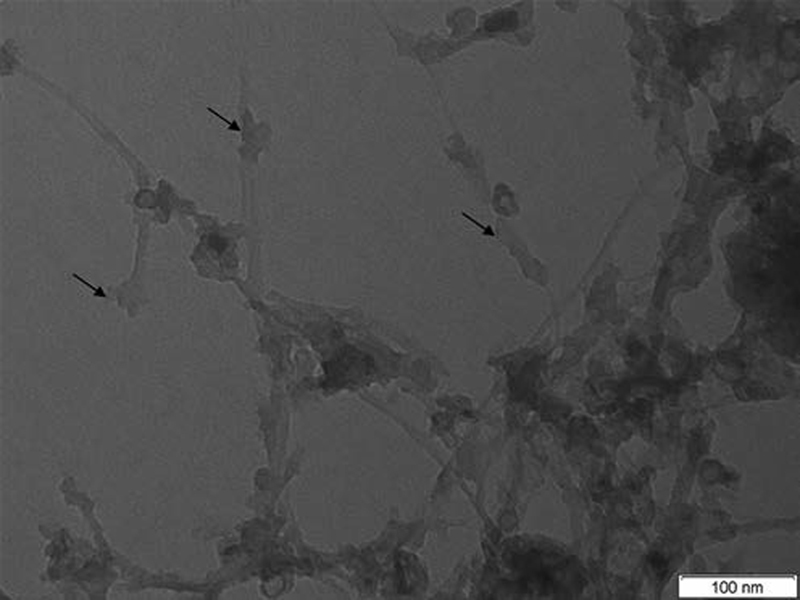
Transmission electron microscopy image of SWCNTs after silanization (SWCNTs/SiO _2_ -ATES). The nanotubes appear scattered and the arrows show the growth of nano-particles of silicon oxides adhered to their surfaces. SWCNT, single-wall carbon nanotubes.

As refering to the flexural strength data in Table 1 , no significant difference was found among SWCNTs/0.1, SWCNTs/0.2, and SWCNTs/0.3 groups, comparing to the control group. The results also revealed that the SWCNTs/SiO2-ATES samples showed higher flexural strength than the others experimental groups, with the exception of the unmodified control resin, which exhibited the highest values. The TEM image of the original SWCNTs ( Fig. 2 ), showed that this form contained many impurities, mainly amorphous carbon and metal nanoparticles. The TEM image of SWCNTs after the silanization treatment ( Fig. 3 ) showed that the impurities were almost completely removed, and the dispersion was also improved. The TEM image of SWCNTs/SiO2-ATES also reveals the presence of many spherical nanoparticles that form a thin shell on the outer surface of the SWCNTs.

**Table 1:** Means and standard deviations of the flexural strength(MPa)

Group	CG	SWCNTs/SiO _2_ -ATES	SWCNTs/0.1	SWCNTs/0.2	SWCNTs/0.3
Mean	100.35	91.58	85.24	84.08	83.36
SD	1.25	1.35	1.05	2.04	2.59
	A	B	C	C	C
Abbreviations: SWCNT, single-wall carbon nanotube; CG, Corega group; SD, standard deviation. Note: Horizontally, mean values designated with identical capital letters were not statistically different ( *p* > 0.05).					

## Discussion

In this study, the flexural strength was evaluated to determine the efficacy of indirect composite reinforcement of Sinfony by SWCNTs with two chemical surface modifications. Two main problems have to be solved to effectively improve the material properties of composite by adding CNT as reinforcement. These problems are the interfacial bonding and the dispersion of CNTs in the polymeric matrix. CNTs are an inherently inert material and easy to agglomerate. In addition, slipping in the bundle and weak interface bonding result in inefficient load transfer, decreasing the mechanical properties of CNT reinforced composite. Thus, the particles need some surface modification to reduce clustering and improve their dispersion throughout the composite matrix. 23 This process involves attachment of functional groups to nanotubes walls or covalent attachment. 22

Table 1 shows that the composite containing SWCNTs/SiO2-ATES exhibited higher flexural strength than that of original SWCNTs content 0.1, 0.2, and 0.3 wt%. This lower value of flexural strength obtained was probably caused by a SWCNTs agglomeration, leading to less energy dissipating in the system under viscoelastic deformation. Another explanation may be related to the opacity of the SWCNTs agglomerates. This could have interfered with the degree of conversion of monomers and consequently affected the properties of the composite material. It is more important to have dispersion and an accurate load transfer between nano-reinforcement and matrix than to increase the content of nanotubes. 24 However, when these values were compared to those from composite without nanotubes (CG), the flexural strength of the SWCNTs/SiO2-ATES was consistently lower. In the present study, purified SWCNTs with carboxylic sites were used, and the presence of carboxylic acid groups on the nanotube surface facilitates further functionalization because this group can participate in a variety of chemical reactions. 25 The organosilane ATES, used in this study, supports the combination between Si-OH and OH- of the surface of SWCNTs while the organic portion can react with the composite monomer via the C=C group. 23

When comparing the results of the present study to Zhang et al, 22 some differences can be found. The authors incorporated a silanized SWCNTs, in a weight ratio of 0.1 (SWCNTs: paste), in a composite paste (Durafill; Heraeus-Kulzer, Germany) and reported that the specimens containing modified SWCNTs exhibit considerable improvement in flexural strength compared to Durafill without SWCNTs. This contradictory finding may be attributed to the differing methodologies and composite employed. The authors incorporated a silanized SWCNTs in a weight ratio of 0.1 in a composite paste, using a blended monomer of urethane dimethacrylate. A low viscosity of a resin enables particles to be organized within the resin.

The indirect composite system Sinfony is a microhybrid material that was developed to be applied using the layering technique because of its flow consistence. In this study, the SWCNTs were spatulated directly with the resin paste. This process could have affected the properties of the resin where a large amount of filler had been inserted in an inadequate quantity of organic matrix, resulting in a decrease of the flexural strength of Sinfony resin modified by SWCNTs. Nanotubes must be uniformly dispersed as isolated nanotubes individually coated with polymer.

In other study, authors reported that composites including silanated nanofiller, as opposed to untreated nanofiller, not only presented superior mechanical properties, but were more resistant to water sorption and general wear. 26 These finds are in agreement with our study where SWCNTs/SiO2-ATES presents higher flexural strength than SWCNTs/0.1, SWCNTs/0.2, and SWCNTs/0.3 groups that are not silanized. Moreover, TEM images of SWCNTs after silanization showed the presence of growth of nanoparticles of silicon oxides adhered to the surfaces ( Fig. 2 ). Despite these analysis have confirmed that SWCNTs were silanized, the process utilized might not be so effective because CG present higher values of flexural strength when compared with SWCNTs/SiO2-ATES group, suggesting that the SWCNTs act as​​ weakness area where there is a poor chemical bonding between the nanofiller and Sinfony organic phase. This hypothesis may also be suggested for SWCNTs/SiO2-ATES when compared with SWCNTs/0.1; SWCNTs/0.2, and SWCNTs/0.3 groups. Within the limitation of this study, the results showed that the incorporation of CNT could be an interesting procedure to improve dental composites flexural strength; however, this incorporation should be performed with caution, since the amount of CNT can interfere in the wettability of the resin matrix and the degree of conversion. Further studies should investigate different surface treatments of CNTs and the effects of its incorporation on a dental composite.

## Conclusion

The silanization process improves the SWCNTs strength proprieties, but the modification of chemical bonding between SWCNT and SWCNT/SiO _2_ -ATES modified resins, in different concentrations, did not improve the composite resin flexural strength. Since RBC restorations are widely used, the clinical indications of each type of resin composite are dictated by the amount of stresses applied on the restoration as well as the esthetic requirements. Therefore, understanding how CNT reinforces dental composites can be an alternative to create a stronger and cheap material that could be used in the dental office in cases of fixed partial dentures.
